# Inequalities in Temporal Effects on Cervical Cancer Mortality in States in Different Geographic Regions of Brazil: An Ecological Study

**DOI:** 10.3390/ijerph19095591

**Published:** 2022-05-05

**Authors:** Karina Cardoso Meira, Carinne Magnago, Angelo Braga Mendonça, Stephane Fernanda Soares Duarte, Pedro Henrique Oliveira de Freitas, Juliano dos Santos, Dyego Leandro Bezerra de Souza, Taynãna César Simões

**Affiliations:** 1 Health School, Federal University of Rio Grande do Norte, Natal 59078-970, Brazil; 2 School of Public Health, University of São Paulo, São Paulo 01246-904, Brazil; carinne@usp.br; 3 Brazilian National Cancer Institute, Rio de Janeiro 20230-130, Brazil; angeloprimax@gmail.com (A.B.M.); jlnsantos@yahoo.com.br (J.d.S.); 4 Department of Demography and Actuarial Sciences, Federal University of Rio Grande Norte, Natal 59078-970, Brazil; fersoa@ufrn.edu.br (S.F.S.D.); pedrofreitasufrn@gmail.com (P.H.O.d.F.); 5 Public Health Department, Federal University of Rio Grande do Norte, Natal 59078-970, Brazil; dysouz@yahoo.com.br; 6 René Rachou Institute, Oswaldo Cruz Foundation, Belo Horizonte 30190-002, Brazil; taynana.simoes@fiocruz.br

**Keywords:** uterine cervical neoplasms, mortality, age–period–cohort analysis, forecasting, Brazil

## Abstract

Cervical cancer is a public health issue with high disease burden and mortality in Brazil. The objectives of the present study were, firstly, to analyze age, period, and cohort effects on cervical cancer mortality in women 20 years old or older from 1980 to 2019 in the North, South, and Southeast Regions of Brazil; and secondly, to evaluate whether the implementation of a national screening program and the expansion of access to public health services impacted the examined period and reduced the risk of death compared with previous years and among younger cohorts. The effects were estimated by applying Poisson regression models with estimable functions. The highest mortality rate per 100,000 women was found in Amazonas (24.13), and the lowest in São Paulo (10.56). A positive gradient was obtained for death rates as women’s age increased. The states in the most developed regions (South and Southeast) showed a reduction in the risk of death in the period that followed the implementation of the screening program and in the cohort from the 1960s onwards. The North Region showed a decreased risk of death only in Amapá (2000–2004) and Tocantins (1995–2004; 2010–2019). The findings indicate that health inequities remain in Brazil and suggest that the health system has limitations in terms of decreasing mortality associated with this type of cancer in regions of lower socioeconomic development.

## 1. Introduction

Cervical cancer (CC) mortality is considered preventable because there are well-defined steps in the natural history of this disease. Additionally, a secondary prevention measure that shows high efficacy and effectiveness in identifying precursor lesions and initial stages of the disease, the Pap test, has been available for over half a century [1,2]. In developed countries, the implementation of organized screening programs, with high coverage and quality, has been associated with a marked reduction in the incidence and mortality of this disease [3,4,5,6,7]. In the 2000s, vaccines against the human papillomavirus (HPV), which is directly related to CC, were developed and adopted in many countries [8,9]. Despite all these preventive measures, this type of cancer has the fourth global highest incidence in women, with 604,000 new cases and 342,000 deaths estimated for 2020. It is more frequently diagnosed in developing countries, especially those in sub-Saharan Africa, where mortality rates are up to 18 times higher than those in Australia and New Zealand [1].

In Brazil, until the first half of the 1990s, when the Brazilian Unified Health System (SUS) was at the beginning of its implementation, CC control actions in Brazil were very sporadic. They began to be the focus of public policies only in the 2000s, with the implementation of the National Program against Cervical Cancer (PNCC, as per its initialism in Portuguese), which had the objective of expanding early screening by means of the Pap test. In the years that followed, actions oriented toward preventing and controlling CC were extended to all Brazilian states as a consequence of the expansion of the primary health care (PHC) network and the establishment of the National Cancer Care Policy (PNAO, as per its abbreviation in Portuguese) [10].

These initiatives succeeded in increasing women’s access to the Pap test, whose current coverage is higher than 80%, but with important variations among the five regions (79.0% in the North, 76.4% in the Northeast, 78.8% in the Center-West, 84.8% in the South, and 84.1% in the Southeast) and the states [11]. Despite the increase in Pap Test coverage in all Brazilian states, important disparities in incidence and mortality from this neoplasm persist between states with greater and lesser socioeconomic development.

Cervical cancer is currently the third most frequent type of cancer in Brazilian women (7.4%), with an estimated 16,590 new cases for each year of the 2020–2022 triennium and a probability of 15 cases per 100,000 women. However, it had the second highest incidence in the least developed regions (North, Northeast, and Midwest, with probabilities of 21.20, 17.62, and 15.92 cases per 100,000 women, respectively), the fourth highest in the South (17.48 cases per 100,000 women), and the fifth highest in the Southeast (12.01 cases per 100,000 women). The disparities between states in the same region are even more alarming, with the incidence coefficient per 100,000 women ranging from 17.22 to 33.50 in the North, and from 5.93 to 13.67 in the South and Southeast [12].

The persistence of inequalities related to socioeconomic conditions and access to health services has also resulted in lower coverage of the Pap test and higher levels of CC mortality in women who are older, black, indigenous, have a low level of education, do not have health insurance, or live in the least developed regions of Brazil [11,13,14,15,16,17,18,19,20,21,22,23,24]. This scenario has proven persistent even after the socioeconomic and health advances that occurred in Brazil from the 2000s onwards, which ended up increasing the inequalities between high- and low-income areas, as is usual in developing countries [25,26,27].

The evolution of CC incidence and mortality rates can be correlated with both prevalence of risk factors in the female population (such as use of oral contraceptives, fertility rates, age at sexual initiation, and number of sexual partners) and access to quality screening programs and timely treatment, including surgery, chemotherapy, and radiation therapy [3,4,5,6,26]. It is known that these factors are not evenly distributed over all age groups, periods, and cohorts. Therefore, it is necessary to use age–period–cohort (APC) models to understand the behavior of this pathology over time [3,4,5,6,28,29,30,31].

The age effect consists of changes in the risks of developing diseases and dying that are compatible with the biological alterations that occur in individuals as they age. Older populations tend to show higher incidence, prevalence, and mortality associated with chronic noncommunicable diseases, whereas people in younger age groups are more susceptible to sexually transmitted infections, homicides, and transport-related accidents. The temporal effect consists of structural transformations that affect all age groups simultaneously, such as large sociocultural, economic, and environmental changes. Some examples are major wars, pandemics, economic crises, and health policies. Members of distinct cohorts experience the impact of these changes differently over their lives and, therefore, exposure to risk and protective factors in disease development varies according to age group [29,30,31].

By taking into account the health inequities between Brazilian states regarding CC mortality, the implementation of PNCC, and the expansion of PHC in Brazil over the past twenty years, the present study aims to analyze age, time, and birth cohort effects on CC mortality in women 20 years old or older from 1980 to 2019 in the North, Southeast, and South Regions of Brazil, as well as assessing whether the implementation of a national screening program and the expansion of access to health services promoted temporal effects and reduced the risk of death over the past years and in younger cohorts.

## 2. Materials and Methods

### 2.1. Study Design, Study Population, and Location Characterization

This was a temporal trend ecological study in which the settings were the North, South, and Southeast Regions of Brazil. The Guidelines for Accurate and Transparent Health Estimates Reporting were applied [32].

**Figure 1:**
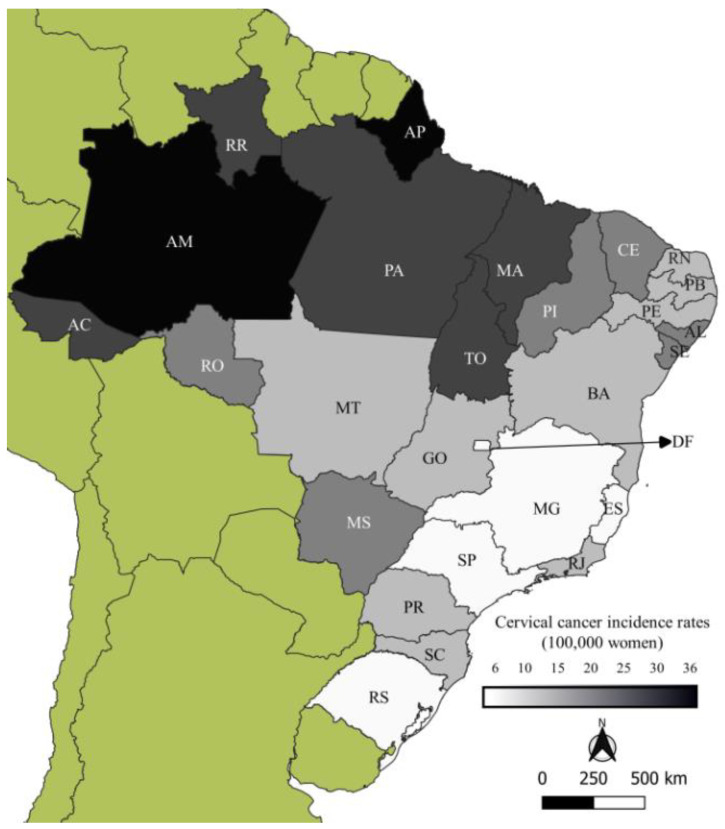
Cervical cancer incidence per 100,000 women in Brazilian states (2020). Source: Prepared by the authors with data from Brazilian National Cancer Institute [12].

Brazil has 26 states and one federal district, which are grouped into five macro regions with similar geographic, cultural, and socioeconomic characteristics. The most developed regions, which show better health indicators, are the South, made up of the states of Paraná (PR), Santa Catarina (SC), and Rio Grande do Sul (RS), and Southeast, whose states are Espírito Santo (ES), Minas Gerais (MG), Rio de Janeiro (RJ), and São Paulo (SP). The North Region includes the states of Acre (AC), Amazonas (AM), Amapá (AP), Pará (PA), Rondônia (RO), Roraima (RR), and Tocantins (TO). These states are in the Amazon Forest area and have low demographic density, high socioeconomic vulnerability, and high CC incidence [12,33] (Figure 1, and Table S1 in Supplementary Material).

The South and Southeast regions have higher concentrations of high-complexity cancer care units and show the highest values for the health component of the Firjan Index of Municipal Development (Table S2 in Supplementary Materials). This index evaluates economic development in three fields: work, education, and health. The health component assesses health conditions by means of the following items: proportion of adequate prenatal care, proportion of registries of deaths whose causes were classified as poorly defined, number of children’s deaths from preventable causes, and number of hospitalizations whose causes were PHC-sensitive, that is, that were caused by complications of health problems that can be managed by PHC services. The index ranges from 0 to 1 and indicates the level of development, overall and by component, according to four categories: low (from 0.0 to 0.39); reasonable (from 0.40 to 0.59); moderate (from 0.60 to 0.79); and high (from 0.80 to 1.0) [34].

### 2.2. Study Variables

The records used in the present study were extracted from the SUS Informatics Department database (DATASUS) [35], which gathers and systematizes data from other information systems, including those from the Brazilian Institute of Geography and Statistics (IBGE) and the Brazilian Mortality Information System (SIM). The study population consisted of women aged 20 years or more. Population data were obtained from the Brazilian Institute of Geography and Statistics (IBGE), based on the population censuses of 1980, 1991, 2000 and 2010. For the intercensus years, estimates were used, also made available by the IBGE.

The Brazilian Mortality Information System gathers death records for all Brazilian states and municipalities in the period between 1970 and 2019. The microdata are available as dbc files that can be converted into the dbf extension by using Tabwin (version 4.15 for Windows), software that is provided by the Brazilian Ministry of Health (Brasília, Brazil) [35]. After conversion of the data into the dbf format, the death records for each state for each year from 1980 to 2019 were grouped by using R software (version 4.1, R Core Team, Vienna, Austria, 2018) [36]. The records were then extracted for women 20 years old or older whose basic cause of death, according to the International Statistical Classification of Diseases and Related Health Problems (ICD-9 and ICD-10), was cervical cancer (180 and C53).

Due to the disparities observed in the quality and coverage of death records between the locations under study [37,38,39,40,41], the authors of the present study opted to use correction techniques to make up for possible limitations [13,14,20]. The correction process was executed independently by two of the authors (KCM and SFSD) and was confirmed by a third author (PHOF), and included the following steps:

(i) proportional redistribution of 50% of deaths classified as ill-defined cause among defined natural causes [13,14,20,42], stratified by the state, age group and year of death.
(ii) the proportional redistribution classified as incomplete diagnosis among all cancers and the deaths classified as incomplete diagnosis of female genital tract cancer, by age group, year of death and state (184, 195, 196, 197, 198, 199, C57, C76, C77, C78, C79, C80, and C97);
(iii) the proportional redistribution of deaths classified as unspecified uterine cancer relating to cancers of the uterus (179, and C55), cervical cancer (189 and C53) and endometrial cancer (182 and C54), according to age group, year of death and state.

The sum of the values obtained in the previous steps (i, ii and iii) was added to the cervical cancer deaths registered in SIM/DATASUS; and finally, a correction in death coverage (underreporting), using the correction factors proposed by Queiroz et al. (2017) [39], for females by Brazilian state for the 1980s, 1990s, 2000s and 2010s. At this stage, the correction factors for each decade were multiplied by the number of deaths obtained in Step iv. After correction, the age groups and periods were grouped into five-year intervals. Age groups from 20–24 years to 80 years or more had their excess zeros in the older groups evaluated, which resulted in I = 13 age groups, J = 8 quinquenniums, and K = I + J − 1 = 20 birth cohorts, ranging from 1900 to 1995 [21,22], where i = 1, …, I; j = 1, …, J; k = 1, …, K; and K = I + J − 1. Since the existence of the state of Tocantins came into force in 1989, the APC effect was evaluated between 1995 and 2019. Therefore, there were five periods, 13 age groups, and 17 cohorts (1915 to 1995).

Mortality rates by age group, period, and location were calculated for five-year intervals. Coefficients standardized by the direct method were also estimated by using the standard population proposed by Segi (1966) and modified by Doll and Hill [43]. Once the average rates by period and state were obtained, thematic maps by quinquennium were produced using QGIS version 3.1.6, a user-friendly open-source geographic information system licensed under the GNU General Public License [44]. The captions show the quintiles.

### 2.3. Statistical Analysis

The APC effects were calculated by applying the Poisson regression model, which assumes that the number of deaths followed a Poisson distribution. In this model, the effects act on the rate in a multiplicative way and, therefore, the logarithm of the expected value of the rate is a linear function of the age, period, and cohort effects [30,31].

$$\ln\left(E\left[r_{\mathrm{ij}}\right]\right)=\ln\left(\frac{\theta_{\mathrm{ij}}}{N_{\mathrm{ij}}}\right)=\mu +\alpha_{i}+\beta_{j}+\gamma_{k},$$

where $E\left[r_{\mathrm{ij}}\right]$ is the expected mortality rate at age i and period j; $\theta_{\mathrm{ij}}$ indicates the number of deaths at age i and period j; $N_{\mathrm{ij}}$ is the population at risk of death at age i and period j; µ is the average rate; $\alpha_{i}$, β_j_, and $\gamma_{k}$ are the effects of age group i, period j, and cohort k, respectively [29,30,31].

The parameters were estimated by using the approach proposed by Holford [29], which limits analysis of the effects to linear combinations and curvatures that remain constant despite the parameterization used [28,29,30]. The linear trend of the effects has two components: the first is the linear age effect, and the second is called drift (the linear trend of the period and cohort effects). The sum of the curvature of age and period $\left(\alpha_{L}{+\beta }_{L}\right)$ is the age longitudinal trend, in which $\alpha_{L}$ and $\beta_{L}$ are the linear age and period trends, respectively. The linear trend of the logarithm of the specific age rates is the drift, which is the sum of the curvatures ($\beta_{L}{+\gamma }_{L}$), where $\beta_{L}$ and $\gamma_{L}$ are the linear period and cohort trends, respectively [30,31].

The analyses shown in the present study used the interval from 1995 to 1999 as the reference period, since it preceded the implementation of PNCC (1998) and PNAO (2005). The reference cohort was for the period from 1950 to 1954 because central cohorts tend to be more stable and complete than first and last cohorts [30,31].

Model fitting was assessed by using deviance, defined as twice the logarithm of the likelihood function of the complete model divided by the logarithm of the likelihood function of the estimated model. The contribution of the effects was evaluated by comparing the deviance of the model with a specific effect to the deviance for the complete model (APC). Results with *p* ≤ 0.05 were considered statistically significant.

The risk of death for periods and cohorts was estimated as relative risk (RR) and its respective 95% confidence intervals (CI). The probabilistic models used to estimate the effects were taken from the Epi library, version 1.1.18 (R Foundation for Statistical Computing, Vienna, Austria, http://www.r-project.org; accessed on 20 April 2020) of the R program (version 4.1) (R Core Team, 2018) [36].

## 3. Results

There was a percentage increase in corrected CC mortality rates in comparison with uncorrected rates in all the states, especially in the 1980s and the 1990–1994 period. In the South and Southeast regions, this increase ranged from 60.14% (ES) to 89.30% (MG). The North Region had the highest average rates before and after the correction and showed an increase that varied from 45.13% (AM) to 83.95% (PA). In the South and Southeast regions, the highest corrected rate per 100,000 women was found in the state of Espírito Santo (14.67 deaths), and the lowest in SP (10.53). These coefficients were considerably lower than those obtained for the North Region, whose lowest and highest rates were those calculated for Rondônia (17.91), Amazonas (35.03), Amapá (35.02) and Roraima (30.02), respectively. The ratio of average CC mortality rates in the states in the South and Southeast regions to the number in the state of Amazonas was over 2.0, ranging from 2.39 (ES) to 3.33 (SP) (Table 1 presents the corrected rates. Uncorrected rates are shown in Table S3 in Supplementary Materials).

In all the states, the correction step that led to the highest increase in the number of deaths was the proportional redistribution of the records classified as UCOUP. The death records classified as UCOUP showed a proportion between uterine cancer death records (CC + EC + UCOUP) ranging from 5.01% to 56.52% between the states and five-year periods studied. In general, there was a reduction in the frequency of UCOUP in the 2000s (Table S4 in Supplementary Material).

In all the periods and states, UCOUP mortality rates were higher than those associated with endometrial cancer (EC), with a reduction in the rates for records classified as UCOUP over time. The UCOUP coefficients were higher than the CC coefficients in some periods, with the numbers for the state of Minas Gerais between 1980 and 1994, the state of Rio de Janeiro from 1985 to 1994, and the state of Rio Grande do Sul in the 1980–1984 and 1990–1994 periods standing out (Table S5 in Supplementary Materials).

In all the analyzed periods, the highest rates were obtained for the North Region, and the lowest for the Southeast Region. The mortality rates decreased in the South and Southeast regions from the 1995–1999 period onwards. In the states in the North Region, there were three temporal evolution patterns in this period: increases in Amazonas and Roraima, stability in Pará and Tocantins, and decreases in Amapá, Acre, and Rondônia (Table 1).

The average cervical cancer mortality rates by age group showed a progressive increase as women got older in all the locations, and the highest mortality coefficients were found for the states in the North Region (Table 2).

**Figure 2:**
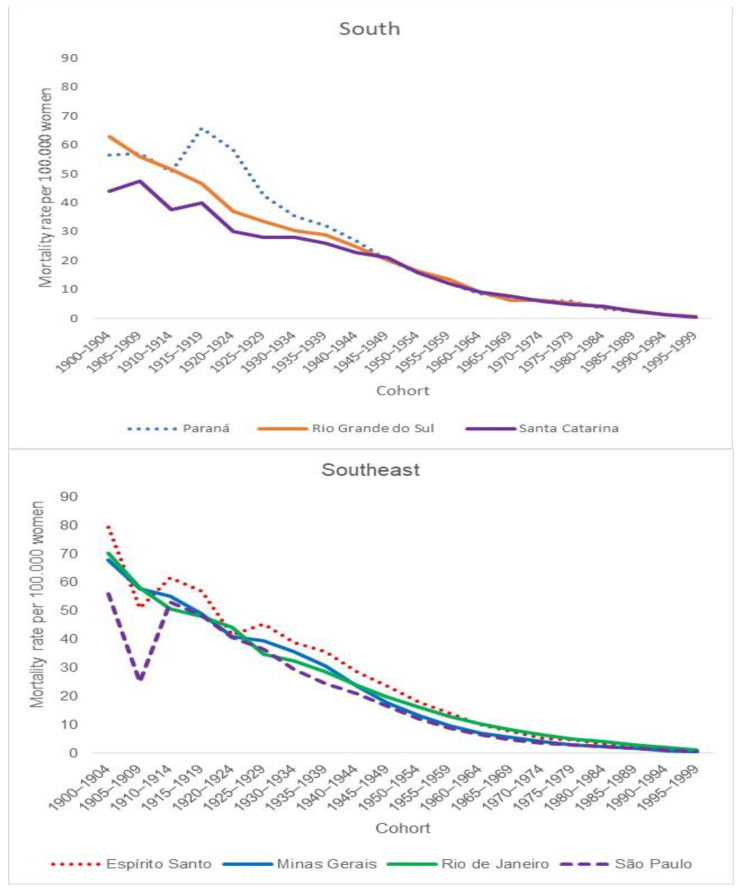
Average cervical cancer mortality rates by cohort in the Brazilian states in the South and Southeast Regions.

**Figure 3:**
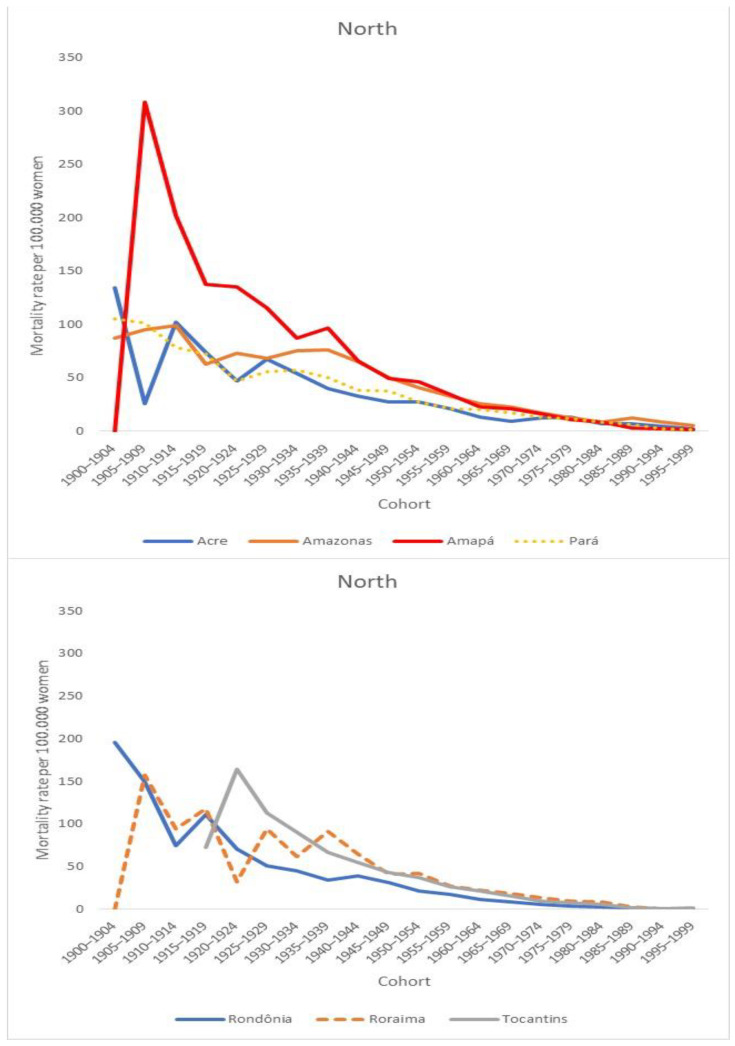
Average cervical cancer mortality rates by cohort in the Brazilian states in the North Region.

Regarding average rates by cohort, there was a progressive decrease in the coefficients for younger cohorts. The states in the North Region showed higher coefficients in comparison with those calculated for the states in the South and Southeast regions (Figure 2 and Figure 3).

After fitting the probabilistic models by using estimable functions, the complete model (APC) was the one that best fits the data in all the states (Tables S6 and S7 in Supplementary Materials).

The linear age trend combined with period (age–drift) shows a decrease in all the states in the South and Southeast regions and in the states of Amapá and Rondônia, located in the North Region; however, there was an increase for AM and stationarity for Acre, Pará, Roraima and Tocantins (Table 3). The age–drift trend is considered stationary when the 95% CI contains the value 1, descending when the trend and the 95% CI are lower than 1, and ascending when the trend and the 95% CI are higher than 1.

**Figure 4:**
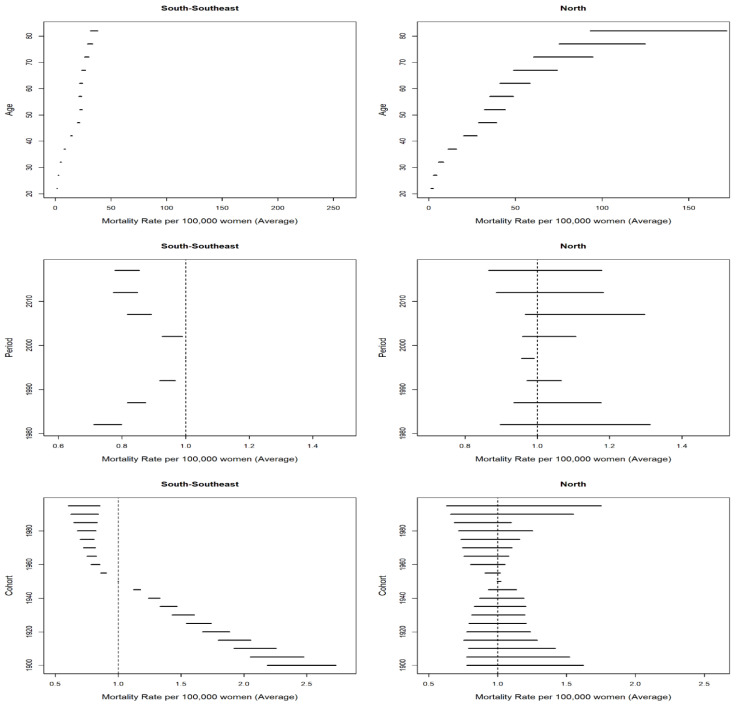
Results of the effect of age, period and cohort adjusted for cervical cancer mortality in the North, South, and Southeast regions of Brazil.

Analysis of the temporal effects by region showed the existence of the highest average age rates in the North Region, reductions in the risk in the two periods in the 2000s in the South and Southeast regions, nonsignificant effects in the North Region, and decreases in the death probability in the cohorts from the 1960s onwards in the South and Southeast regions (Figure 4).

**Figure 5:**
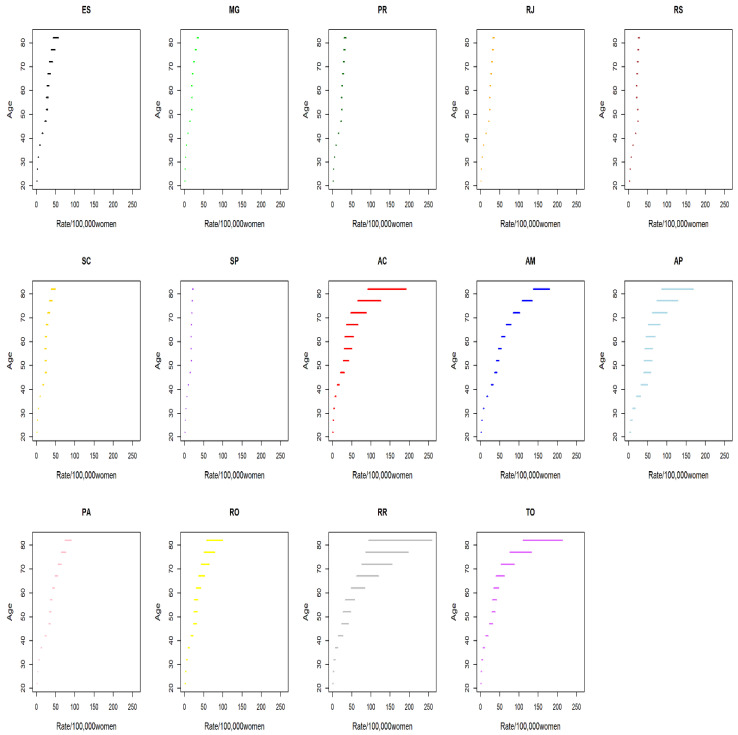
Results of the age–period–cohort model adjusted for mortality from cervical cancer according to the age effect in the states in the North, South, and Southeast Regions (1980–2019). AC = Acre; AM = Amazonas; AP = Amapá; PA = Pará; RO = Rondônia; RR = Roraima; TO = Tocantins; ES = Espírito Santo; MG = Minas Gerais; RJ = Rio de Janeiro; SP = São Paulo; PR = Paraná; RS = Rio Grande do Sul; SC = Santa Catarina.

The average CC mortality rates by age group fitted for period and cohort effects showed a positive gradient in all the locations as women got older, with the highest values found in the North Region (Figure 5).

**Figure 6:**
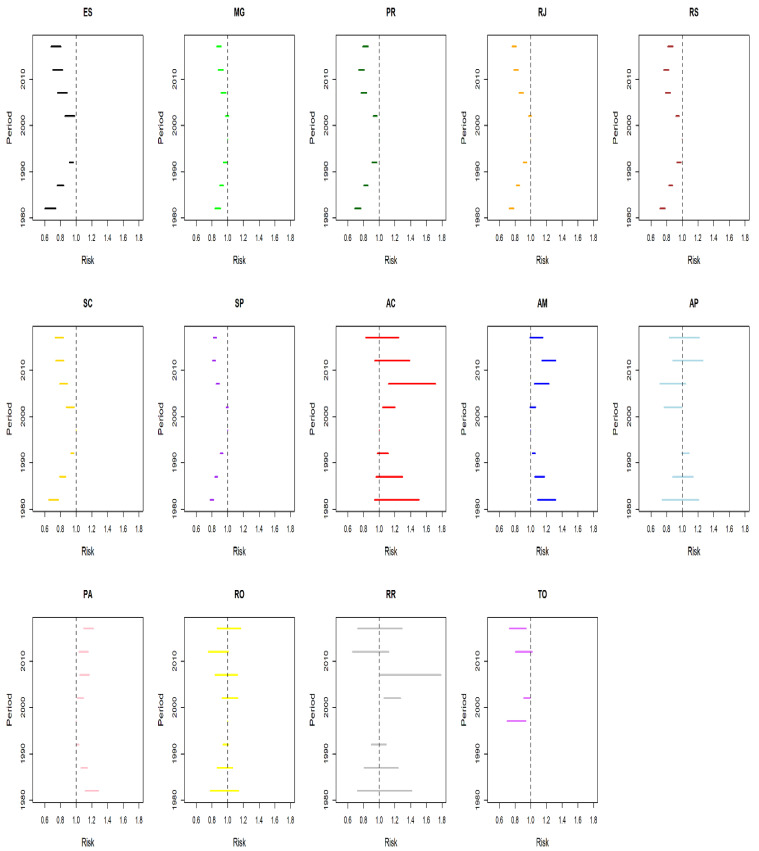
Results of the age–period–cohort model adjusted for mortality from cervical cancer according to the period effect in the states in the North, South, and Southeast regions in Brazil (1980–2019). AC = Acre; AM = Amazonas; AP = Amapá; PA = Pará; RO = Rondônia; RR = Roraima; TO = Tocantins; ES = Espírito Santo; MG = Minas Gerais; RJ = Rio de Janeiro; SP = São Paulo; PR = Paraná; RS = Rio Grande do Sul; SC = Santa Catarina.

Regarding risk of death from CC by period fitted by age and cohort effects, if the 1995–1999 quinquennium is considered as a reference, there were reductions (RR < 1) in all the quinquenniums in the states in the South and Southeast regions, except for the 2000–2004 period in the states of Rio de Janeiro and São Paulo, where the decreases were not statistically significant. In the North Region, there was an increase in the following states and periods: Acre and Roraima in 2000–2009; Amazonas, in all the periods but 2000–2004 and 2015–2019; and Pará, in all the quinquenniums, excluding 1980–1984. Reductions in risk were observed in Amapá in 2000–2004 and Tocantins in 1995–2004 and 2015–2019. In Rondônia, the period effect was not significant in any period (Figure 6).

**Figure 7:**
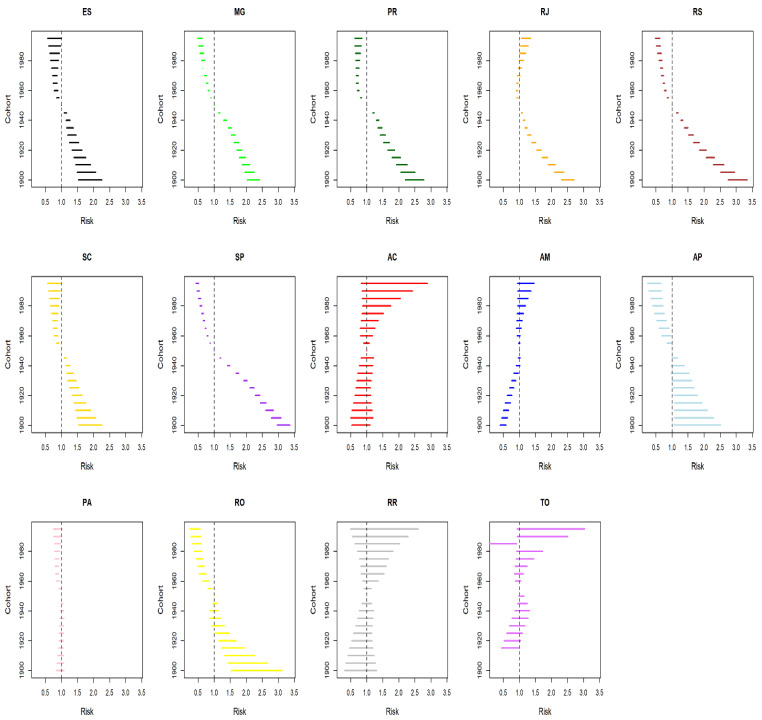
Results of the age–period–cohort model adjusted for mortality from cervical cancer according to the cohort effect in the states in the North, South, and Southeast regions in Brazil (1980–2019). AC = Acre; AM = Amazonas; AP = Amapá; PA = Pará; RO = Rondônia; RR = Roraima; TO = Tocantins; ES = Espírito Santo; MG = Minas Gerais; RJ = Rio de Janeiro; SP = São Paulo; PR = Paraná; RS = Rio Grande do Sul; SC = Santa Catarina.

Analysis of the cohort effect on CC mortality fitted for the age and period effect showed that when the 1950–1955 quinquennium is taken as a reference, the RR of death increased (RR > 1) for older cohorts and decreased for younger cohorts (RR < 1) in all the states in the South and Southeast regions, except for the 1985–1995 cohort in the state of Rio de Janeiro (RR > 1, *p* < 0.001) (Figure 7). A similar outcome was found for the states of Amapá, Pará, and Rondônia. Acre, Amazonas, Roraima, and Tocantins had reductions in risk for women in older cohorts and increases for women in younger cohorts, but with no statistical significance (Figure 7).

## 4. Discussion

In this study, disparities were observed in the quality and coverage of death records, magnitude of cervical cancer mortality rates, time trend, and APC effect according to socioeconomic development in the federative units of Brazil in the study. In the states of the North region, there was worse quality and lower coverage of mortality records in the Mortality Information System; in addition, these regions showed higher mortality rates relating to the states of the South and Southeast. Furthermore, the states in the most developed regions (South and Southeast) showed a reduction in the risk of death in the period that followed the implementation of the screening program and in the cohort from the 1960s onwards. However, in the states of the North region, there was a reduction in the risk of death in only two states (Amapá and Tocantins).

Regarding the quality and coverage of death records, the present study verified an increase of over 60% in mortality rates after the process of correction of the death records in all the analyzed places. However, worse record quality was found in the states in the North Region and in Minas Gerais, especially in the 1980s and 1990s. The high proportion of records classified as UCOUP was noteworthy, and their redistribution was the main factor responsible for the increase in the number of deaths from CC. These findings corroborated the results of other Brazilian studies that have indicated changes in the temporal trend in capitals and municipalities in the interior of the states after evaluation of corrected and uncorrected records of death from CC. The latter have indicated an increase in mortality in capitals and a decrease in the interior of the states, whereas the opposite occurred when time evolution used the corrected number of deaths [13,14,15,20,45,46]. Similarly, after the correction of death records in the states in the Northeast Region from 1980 to 2016, a marked increase in mortality was found in the states with the worst socioeconomic conditions, Maranhão and Piauí, especially in the 1980s and 1990s [20].

Despite the reduction in the number of records classified as UCOUP from the 2000s onwards in the analyzed areas, the proportion of deaths from this cause remains high, especially in elderly women, with numbers higher than those for endometrial cancer, even in places with higher socioeconomic development. These results suggest a low level of diagnostic problem-solving capacity in the Brazilian health system, and also that part of the female population does not have access to, or does not adhere to, the screening program [13,18,19,20,21,22,47,48]. Although Pap test coverage has increased in Brazil, it remains lower than 85% in all the regions, with the worst indexes in the places with the lowest socioeconomic development [11]. Additionally, the frequency of carrying out the test is irregular, and there are limitations in access to diagnostic services, which often results in late CC diagnosis Another point is that the diagnosis does not always ensure that treatment will be initiated and continued, since there are also limitations in access to specialized cancer services, which are also concentrated in the most developed areas. Therefore, a considerable percentage of women under cancer treatment receive care in municipalities or even states other than those where they live [48,49].

Cervical cancer mortality also remains high, although it decreased in nearly all the North, South and Southeast states over 1980–2019. Higher rates were observed in the states in the North Region, whose values were similar to those found in countries with no universal or free screening programs [1] and in Brazilian states with the worst socioeconomic indicators, such as Maranhão and Piauí, located in the Northeast Region [20]. Brazil has been through a process of settlement and exploitation that has produced historical regional inequalities that have continued until the present, with the concentration of production activities and economic hubs in the South and Southeast regions and the coast of the Northeast Region [26,27]. These disparities often extend into the health field and are noticeable in the unequal health conditions of different populations, levels of vulnerability and exposure to risk of developing diseases and dying, and the special access to resources and services available in the health system [50]. The SUS configuration itself expresses and reproduces these inequalities: historically, PHC services expanded into the poorest regions of the country, whereas medium and high-complexity services and equipment were concentrated in capitals and metropolises, notably those in the center and south of Brazil. This pattern of distribution of care offered, and fragmentation, results in geographic and access inequities and great variation in the flow of patients for the use of services [25,49,50].

The insufficient number of services for the prevention, diagnosis, and treatment of CC in a timely manner in the states of the North region, together with their geographical characteristics of large territorial extensions, whose internal transport occurs largely by rivers, makes access to health care difficult for the riverside population and indigenous community [49,50]. Added to this reality, the ethnic composition of the population is predominantly black and indigenous—ethnic groups that historically have less access to work, income, education, and health services when compared with the white population. Standing out, the states of Amapá (4.84%) and Roraima (11.01%) have the highest proportion of indigenous population in Brazil, according to the last Census (2010) (Table S1 in Supplementary Materials): these states, together with Amazonas, showed the highest CHD mortality rates in our research.

The present study sought to evaluate whether the existence of a national universal and free screening program for all states of Brazil, that includes early diagnosis by means of oncotic cytology (the Pap test) and early treatment for precursor lesions and cancer, promoted period effects and reduced the risk of death from CC in the two quinquenniums of the 2000s and younger cohorts, who have greater access to health services and cancer treatment. Differences in temporal effects (APC) between the states in the North, South, and Southeast regions were also examined.

Analysis of temporal effects (APC) indicates that there is a positive gradient in mortality rates as women age, after fitting of the APC models by using estimable functions. Similar results have been found in Brazilian studies that analyzed data from the Northeast Region [20], the cities of São Paulo and Rio de Janeiro [51], and the state of Minas Gerais [52]. The age effect is similar to those reported for developed countries (the United States, Canada, the Western European countries, Japan, the United Kingdom, and Spain) and developing countries (Singapore, China, South Korea, Estonia, and the Baltic states) [3,4,5,6,7,53,54,55,56].

The higher CC mortality rates in older women may be related to the physiological alterations resulting from aging [57,58] but could also be related to lower coverage by the gynecologic preventive test. Many women tend to reduce the frequency with which they have gynecologic appointments at the end of their reproductive lives, mostly because policies oriented toward women’s health still focus more on the pregnancy and postpartum periods [11,18,19,20,21,22]. This situation contributes to the increased probability of these women being diagnosed at advanced stages of CC or UCOUP tumors in comparison with what happens with younger women [13,20,23,24].

The present study also found marked differences in temporal effects between the examined regions. There was a reduction in the mortality trend (age-drift) and risk of death in the periods after the implementation of PNCC, in the 2000s, and in cohorts from the 1960s onwards in the states in the South and Southeast regions. Regarding the North Region, the temporal trend (age-drift) decreased in the states of Amapá and Rondônia, increased in the state of Amazonas, and remained stable in the other states. The risk of death by period was reduced in the states of Amapá (2000–2004) and Tocantins (1995–2004 and 2015–2019) only. The other states did not show reductions in mortality rates, and the risk of death increased in the periods after the implementation of PNCC in Acre, Amazonas, Pará, and Roraima.

Increases in incidence and mortality are expected in the first years following implementation of large-scale CC screening and treatment policies, since women who had not previously had access to diagnostic testing begin having it and are diagnosed; some will be at an advanced stage of the disease, for which no therapeutic possibilities exist. In addition, these initiatives tend to improve the process of recording cases and deaths. In time, the trend tends to reverse, given that CC precursor lesions are identified and treated early, a scenario that occurred in Canada, the Scandinavian countries, England, South Korea, and Singapore. In these countries, implementing programs with free access, high population coverage, high quality in the processes of collection, storage, and reading of samples, and guarantee of follow-up into treatment promoted substantial reductions in CC incidence and mortality. These effects were noticeable even in generations that showed higher prevalence of exposure to factors that increase the risk of infection with HPV and persistence of the associated lesions [3,4,5,6,7,9,53,54,55,56]. In contrast, regions that do not have systematized screening plans and face difficulty expanding access to and the number of specialized cancer services showed a growing trend of incidence and deaths, similar to what happens in the Eastern Europe countries (Armenia, Azerbaijan, Kazakhstan, Ukraine, Russia, and Estonia) and the states in the Brazilian Northeast Region [20,55,56,57].

Likewise, the findings of the present study indicate that expanding the coverage of the Pap test and increasing the number of centers for cancer treatment, especially in the states in the South and Southeast regions, may have contributed to reducing the risk of death from CC in these places, as has been reported in previous studies [13,51,52,58]. Before the implementation of the national CC prevention and control initiatives, these regions already had local programs [18,19,21,59,60,61,62], which may have played a role in the decreasing trend of risk of death over the entire analyzed period. Conversely, the national initiatives do not seem to have been sufficient to reverse the upward trends in the states in the North Region. Several factors may explain this scenario. First, the screening test does not reach a considerable percentage of women, and most who do not have access to it live in the regions that have the highest number of people experiencing poverty (the North and Northeast). These regions also have the lowest number of cancer treatment units and equipment necessary to treat advanced CC [63], and patients who live in these regions usually receive the results of the tests more tardily. Additionally, the North Region has the greatest deficit in the number of biopsies compared with the necessary number for follow-up of diagnostic confirmation of precursor lesions and CC: −70% vs. −42% and −58% in the Southeast and South Regions, respectively [64].

In Brazil, social inequalities occur at several levels. There is great disparity between regions, different states in the same region, and different municipalities in the same state. At the municipal level, there are inequalities between the urban and rural environments and between central and peripheral areas. Women who are not white and have a low level of education show the worst health indicators, even in the most developed regions and states [18,19,20,21,22,65]. A study with over 65,000 women from all the Brazilian regions showed that indigenous and black women had a probability of receiving a diagnosis of advanced CC that was 2.4 and 1.2 times higher, respectively, than white women [23]. Other studies have pointed out that the CC mortality trend has risen in women in lower social strata, even in the richest states in the South and Southeast regions [66,67].

These inequities may be related to the differences found in the present study between the cohort effect in places with low, intermediate, and high income after fitting by age and period. The states with better socioeconomic indicators show an increased risk of death in the cohort prior to the 1960s and reduced risk in younger cohorts. Similar outcomes have been found in developed countries and the cities of São Paulo and Rio de Janeiro [3,4,5,6,7,51,52,53,54,55,56]. According to Vacarella et al. [5], the contradictions in period and cohort-related temporal effects on CC mortality between and within countries can be explained by the interaction of the following conditions: (i) presence, time since implementation, and quality of screening programs; (ii) prevalence of risk factors for CC; and (iii) therapeutic innovations for the advanced stage of the disease.

In Brazil, it is possible that women from older cohorts did not experience the period effect resulting from the introduction of CC prevention and control initiatives, implementation of SUS, and increases in access to and the number of health services. In addition, these women had a higher fertility rate and a lower level of education, and consequently were less aware of their bodies and health, as well as of CC and its preventive measures [3,4,5,6,7,23,25,52,53,54,55,56]. Another possible correlated factor is the higher prevalence of comorbidities in the elderly population, which often hinders application of the therapies available to treat the advanced stage of the disease and, therefore, interferes with the prognosis.

In theory, the implementation of health policies should promote period effects in all age groups included in the recommendations (in Brazil, the priority group is women from 25 to 64 years old). However, because there are differences in access to health services depending on age group, a cohort effect may exist on CC incidence and mortality, since exposure to these protective factors differs according to age [5,6,7,9,11,18,19,20,21,22,48,51,52,53,54,55,56,57]. The increase in relative risk (RR) of death observed in cohorts in the 1985–1999 period in the state of Rio de Janeiro deserves attention. It can be conjectured that this effect may be correlated with two main situations: limits in the screening program, which still fails to reach women with higher risk of developing the disease, such as black and indigenous women, and those with a lower level of education and income, as discussed above [18,19,20,21,22,48,49] and changes in the CC histological type [3,5,56,68]. Studies in South Korea, Canada, and Estonia have shown an increase in incidence and mortality in young women diagnosed with adenocarcinoma and a reduction in these rates in women diagnosed with squamous cell carcinoma [3,5,56,68]. Considering that oncotic cytology is less effective in identifying adenocarcinoma, because it is a fast-growing cancer and is located in the cervical canal, this trend may be associated with the increase in the efficacy and efficiency of histological analysis [3,5,9,56,68].

The cohort effects observed in the states of Acre, Amazonas, Roraima, and Tocantins were similar to those found in other states and countries with lower socioeconomic development and/or lack of structured CC prevention and control programs [4,6,20,56,68]. The substantial reduction of the probability of dying for the cohorts from 1900 to 1954 in Amazonas may be related to the lower exposure of women to risk factors for CC in these cohorts, which reduces incidence and mortality in this group even in the absence of prevention and control measures. This situation is the same as that observed in China, Spain, and the United Kingdom before the implementation of national, public, and free CC prevention and control policies [4,56,69].

The increase in risk of death in women born from 1960 onwards in the states in the North Region, although not significant, is a worrying situation that reinforces the correlation between level of socioeconomic development and inequalities in access to health services [46,49,70,71,72]. It was assumed that, after two decades of implementation of PNCC, there would be a reduction in the risk of death in the two last quinquenniums (2010–2014 and 2015–2019) and in younger cohorts. The reason was that, in theory, these cohorts would have experienced greater exposure to the protection factors resulting from the cervical cancer prevention and control policy; however, this did not occur in Amazonas, Acre, Roraima, or Tocantins.

### Limitations and Strengths of the Study

Issues regarding the completeness and quality of death records in the analyzed states was one of the limitations of the present study. Although there were considerable improvements in the 1990s and 2000s, there was still heterogeneity in quality and coverage of death records when the Brazilian regions were compared, with the least developed regions, the North and Northeast, showing more gaps and discrepancies [13,14,20,37,38,39,40,41]. These differences can influence the temporal trend of mortality associated with diseases and health problems, causing a period effect [29,30,31]. The authors tried to compensate for this by applying correction techniques validated by other Brazilian studies, which led to more reliable mortality estimates.

Another issue was the impossibility of evaluating temporal effects according to CC histological type since the Brazilian Mortality Information System does not offer this information. There is evidence indicating the replacement of one histological type of squamous cell carcinoma by adenocarcinoma when screening programs expand their coverage and improve their quality [3,9,56,68]. Consequently, it would be pertinent to assess this temporal trend in the states in the South and Southeast regions.

Additionally, it was not possible to analyze APC effects by race/color, because there are no data about this variable by age group for the period from 1980 to 2019. Given that black and indigenous women have a higher risk of death from CC, it would be relevant to evaluate whether the protective effect in younger women and in periods in the 2000s also reached this highly vulnerable group. Last, the effects of risk and protective factors for CC incidence and mortality as covariables of the probabilistic models were not evaluated explicitly since the necessary information was not available for the combination of age and period.

There is no consensus in the literature about the best method to correct the identification problem that occurs in the estimation of APC models. In the present study, the models were estimated by using estimable functions, which is the most recommended methodology in studies that compare classic statistical methods [31,73]. Despite the mentioned weaknesses, the study showed unpublished and relevant data that may contribute to CC surveillance processes in Brazil. Many studies have assessed the CC mortality temporal trend, but few have corrected death records and analyzed the cohort effect on the mortality profile in several places with marked differences in sociodemographic characteristics and access to health services. The methodology used allowed the development of hypotheses about the contextual factors correlated with the CC mortality temporal trend in 55.5% of the Brazilian states, including the efficacy and effectiveness of PNCC. Consequently, the present study can support evaluation and planning processes in health.

## 5. Conclusions

Analyses by age group and period found the highest average mortality rates in the states in the North Region, and the lowest for the Southeast Region. There was a progressive reduction in the coefficients for younger cohorts in all the states. Analyses of temporal effects showed that the states with higher socioeconomic development had a decrease in the risk of death from CC in the periods that followed the implementation of the national screening program (the 2000s) and in the cohorts after the 1960s. The opposite occurred in the states with the worst Human Development Index and health indicators.

The increase in risk of death in women born from 1960 onwards in states in the North Region, although not significant, is a worrying situation that reinforces the correlation between level of socioeconomic development and inequalities in access to health services. The findings of the present study pointed out the need to adopt health policies that are specific for the North Region and consider its specificities and women’s social and material conditions.

### Policy Implication

Studying the temporal evolution of incidence and mortality rates of diseases and other health problems is important for planning actions in public health and evaluating the implemented policies. Adequate interpretation of morbimortality results is necessary in order to distinguish impacts related to changes in risk patterns over time from those related to population size and age structure. Disease incidence or mortality rates usually vary over time. However, there is no single temporal scale to quantify temporal variability. Time in statistical models is considered to be a replacement or proxy measure for these unobserved processes that contribute to the risk of developing disease. Therefore, it is important to have a basic knowledge of the dynamics of the phenomenon being studied when the choice is made about how to incorporate time into risk estimation models [28,29,30,31].

The present study confirmed the impossibility of evaluating PNCC by using exclusively death records classified as CC, because of the high proportion of records classified as neoplasms in UCOUP. Consequently, it is necessary to consider the temporal trend of mortality by including these two codifications or apply correction techniques that have been documented in the literature since 2010. Analyses based only on records classified as CC suggest that there is a substantial reduction in the magnitude of mortality, when the truth is that the reality is being hidden by the poor quality of the information. The high proportion of deaths associated with tumors in UCOUP indicates the health system’s diagnostic difficulty and weaknesses in reaching certain population groups, especially women who are not white and have a low level of education.

The findings of the present study point out the need to design health policies that are specific for the realities of the states in the North Region, since they have persistently high CC mortality rates. Even after the implementation of the national screening program, there was an increase in risk of death in the 2000s, a fact that is also observed in countries without a universal health system. The North Region has sociodemographic and geographic characteristics that must be taken into account in the planning and implementation of health policies, such as its vast territory, the high number of indigenous and riverside populations, and the logistical difficulties involved in travel by these people into urban areas where health units are located. These characteristics make it impossible to use the same strategies for health education, planning, and PNCC execution that were implemented in the South and Southeast regions.

The specificities of the North Region, however, were not considered in either the current Brazilian Guidelines for CC Screening or the Strategic Action Plan to Tackle Noncommunicable Diseases in Brazil 2011–2022. Policies that standardize public health strategies without considering local and regional specificities can contribute to preserving or increasing health inequalities, including those discussed in the present study.

## Figures and Tables

**Table 1:** Corrected mortality rates from cervical cancer, per 100,000 women in the states in the North, South, and Southeast regions in Brazil.

**South and Southeast**	**1980–1984**	**1985–1989**	**1990–1994**	**1995–1999**	**2000–2004**	**2005–2009**	**2010–2014**	**2015–2019**	SMR *
Paraná	12.81	13.41	14.28	14.93	12.94	10.87	10.35	11.03	14.38
Rio Grande do Sul	18.45	18.40	18.36	17.64	14.04	11.51	9.94	10.19	13.73
Santa Catarina	13.32	13.83	15.71	15.77	13.34	11.66	10.45	10.56	12.33
Espírito Santo	15.55	18.42	18.40	19.86	16.43	14.15	12.10	11.23	14.67
Minas Gerais	16.15	14.90	15.17	13.46	11.46	10.31	8.63	8.20	11.22
Rio de Janeiro	16.48	15.24	16.91	16.20	15.24	12.59	11.83	11.19	13.91
São Paulo	16.64	14.06	15.23	13.96	11.72	8.91	7.42	6.88	10.53
**North**	**1980–1984**	**1985–1989**	**1990–1994**	**1995–1999**	**2000–2004**	**2005–2009**	**2010–2014**	**2015–2019**	**SMR**
Acre	21.48	20.32	27.71	21.77	19.86	17.40	25.23	24.75	22.57
Amazonas	28.71	33.92	25.79	30.89	31.45	37.67	40.14	36.66	35.03
Amapá	40.88	49.16	46.07	38.44	29.94	30.29	33.02	29.96	35.02
Pará	28.25	28.60	25.56	22.99	26.02	26.20	24.89	26.65	26.05
Rondônia	23.80	30.70	20.66	19.44	20.28	16.37	14.18	15.76	17.91
Roraima	33.44	15.94	31.87	24.81	28.46	39.48	25.19	32.35	30.02
Tocantis	_	_	_	19.45	21.31	24.18	22.71	21.12	21.36

* SMR-standardized mortality rate.

**Table 2:** Average cervical cancer mortality rates by age group in the Brazilian states in the North, South and Southeast Region.

Age Group		20 to 24	25 to 29	30 to 34	35 to 39	40 to 44	45 to 49	50 to 54	55 to 59	60 to 64	65 to 69	70 to 74	75 to 79	80 and More Years
States *	AC	0.76	1.04	7.23	11.12	18.63	24.34	33.06	42.92	45.79	42.21	55.16	70.38	104.28
	AM	1.03	4.68	12.09	19.80	32.37	41.82	52.81	53.54	62.42	70.88	94.15	109.74	131.10
	AP	2.47	4.66	9.06	17.60	27.73	41.43	38.22	54.18	59.70	79.32	91.76	152.11	170.89
	PA	0.99	3.30	8.33	14.59	23.24	32.29	36.98	44.50	49.66	56.53	63.99	78.61	90.76
	RO	0.39	1.63	4.46	8.00	12.73	19.47	22.24	28.83	34.74	42.35	53.98	75.37	80.52
	RR	0.60	3.74	6.53	12.21	24.42	30.45	33.57	57.56	50.87	85.59	96.40	109.66	118.51
	TO	0.88	1.99	4.20	10.45	14.97	22.46	28.01	36.29	35.61	49.28	58.66	83.62	118.62
	PR	1.48	2.64	5.25	8.32	11.05	16.51	18.21	21.96	27.28	31.21	42.56	48.19	49.85
	RS	0.56	2.34	5.67	9.81	14.18	18.20	20.19	21.77	23.50	28.08	30.03	33.62	40.98
	SC	0.36	1.94	4.33	8.39	12.60	16.23	18.49	19.66	22.29	22.58	29.26	34.54	38.09
	ES	0.27	1.65	4.07	7.76	11.57	16.18	21.76	24.84	29.53	32.67	40.40	43.80	59.24
	MG	0.24	1.07	2.73	5.09	8.71	12.29	16.37	19.26	22.35	25.94	31.89	38.11	46.20
	RJ	0.44	2.05	4.58	8.09	12.54	17.38	20.69	23.05	26.32	30.07	34.15	38.63	47.23
	SP	0.31	1.21	2.72	5.00	8.44	12.00	15.57	18.86	20.96	24.63	28.89	33.91	36.50

* South, made up of the states of Paraná (PR), Santa Catarina (SC), and Rio Grande do Sul (RS), and Southeast, whose states are Espírito Santo (ES), Minas Gerais (MG), Rio de Janeiro (RJ), and São Paulo (SP). The North Region includes the states of Acre (AC), Amazonas (AM), Amapá (AP), Pará (PA), Rondônia (RO), Roraima (RR), and Tocantins (TO).

**Table 3:** Linear age–drift trend after fitting the APC model by estimable functions for the states in the North, South, and Southeast regions in Brazil.

State	Age–Drift	95% CI	Trend
North			
Acre	1.005	0.998–1.010	Stationary
Amazonas	1.007	1.005–1.010	Ascending
Amapá	0.986	0.980–0.991	Descending
Pará	0.998	0.996–1.000	Stationary
Rondônia	0.982	0.978–0.986	Descending
Roraima	1.002	0.995–1.011	Stationary
Tocantins	1.003	0.996–1.011	Stationary
South			
Paraná	0.980	0.979–0.981	Descending
Rio Grande do Sul	0.978	0.977–0.980	Descending
Santa Catarina	0.989	0.987–0.991	Descending
Southeast			
Espírito Santo	0.984	0.982–0.987	Descending
Minas Gerais	0.981	0.980–0.982	Descending
Rio de Janeiro	0.987	0.986–0.988	Descending
São Paulo	0.975	0.973–0.975	Descending

## Data Availability

The data presented in the present study are available on request from the corresponding author.

## Supplementary Materials

The following are available online at https://www.mdpi.com/article/10.3390/ijerph19095591/s1, Table S1. Sociodemographic characteristics of the states in the North, South, and Southeast Regions of Brazil. Table S2. Health indicators of states in the North, South, and Southeast regions of Brazil. Table S3. Uncorrected cervical cancer mortality rates per 100,000 women in the states in the North, South, and Southeast regions in Brazil.Table S4. Proportion of deaths from uterine cancer of unspecified portion in total uterine cancers, according to states in the North, South, and Southeast regions of Brazil. Table S4. Uncorrected mortality rates from cervical cancer, endometrial cancer, and uterine cancer of unspecified portion per 100,000 women in the states in the North, South, and Southeast regions in Brazil. Table S5. Uncorrected mortality rates from cervical cancer, endometrial cancer, and uterine cancer of unspecified portion per 100,000 women in the states in the North, South, and Southeast regions in Brazil. Table S6. Deviance and *p*-value analysis in sequential construction of age, period, and cohort models for the states in the South and Southeast regions of Brazil. Table S7. Deviance and *p*-value analysis in sequential construction of age, period, and cohort models for the states in the North Region in Brazil.
