# Supplementary material for: Inequalities in Temporal Effects on Cervical Cancer Mortality in States in Different Geographic Regions of Brazil: An Ecological Study
Source: Int J Environ Res Public Health. 2022 May 5;19(9):5591. doi: 10.3390/ijerph19095591 (PMC9105639; doi:10.3390/ijerph19095591)
Supplement: Supplementary file 1 [file ijerph-19-05591-s001.zip › Table S1.pdf]

Table S1. Sociodemographic characteristics of the states in the North, South, and Southeast Regions of Brazil.

Location	HDI (2010)	Life expectancy at birth (2019)	Fertility rate (2019)	MHIPC (2020)[1]	% Black population[2]	% Indigenous population[2]
**North**						
Acre	0.66	74.80	2.39	5,050.18	72.08	2.170
Amazonas	0.67	72.60	2.20	4,720.08	73.93	1.106
Amapá	0.71	74.70	2.20	4,947.22	73.00	4.84
Pará	0.65	72.70	2.05	4,891.82	76.75	0.51
Rondônia	0.69	71.60	1.74	6,476.26	62.47	0.76
Roraima	0.71	72.40	2.14	5,445.82	67.10	11.01
Tocantins	0.70	74.20	1.89	5,872.40	72.24	0.94
**South**						
Paraná	0.75	77.90	1.61	8.354.32	28.25	0.24
Rio Grande do Sul	0.75	78.50	1.55	9.744.86	16.13	0.30
Santa Catarina	0.77	79.90	1.54	9.041.28	15.35	0.25
**Southeast**						
Espírito Santo	0.74	79.10	1.61	7.462.38	56.96	0.26
Minas Gerais	0.73	78.00	1.58	7,279.56	53.49	0.15
Rio de Janeiro	0.76	77.00	1.57	9,545.56	51.69	0.09
São Paulo	0.78	78.90	1.58	10,049.56	34.62	0.10
**Brazil**	**0.72**	**76.60**	**1.69**	**7,645.20**		

Source: Brazilian Institute of Geography and Statistics [32]. HDI: Human Development Index. [1]Monthly household income per capita in US$ (US$ 1 is equivalent to approximately R$ 5.50 – Jan/2022). [2]Resident population, by color or race, according to household status, sex and age, according to the 2010 Census
