# Supplementary material for: Inequalities in Temporal Effects on Cervical Cancer Mortality in States in Different Geographic Regions of Brazil: An Ecological Study
Source: Int J Environ Res Public Health. 2022 May 5;19(9):5591. doi: 10.3390/ijerph19095591 (PMC9105639; doi:10.3390/ijerph19095591)
Supplement: Supplementary file 1 [file ijerph-19-05591-s001.zip › Table S2.pdf]

Table S2. Health indicators of states in the North, South, and Southeast regions of Brazil.

Location	No. of SUS cancer care services (2018)[1]	Child mortality rate (2019)[2]	IFDM (health) (2016)[3]
**North**			
Acre	1.00	15.30	0.59
Amazonas	1.00	16.70	0.56
Amapá	1.00	22.60	0.63
Pará	4.00	15.20	0.59
Rondônia	3.00	18.80	0.76
Roraima	1.00	16.70	0.64
Tocantins	2.00	14.50	0.79
**South**			
Paraná	24.00	8.20	0.86
Rio Grande do Sul	29.00	8.80	0.88
Santa Catarina	17.00	8.40	0.86
**Southeast**			
Espírito Santo	8.00	7.80	0.84
Minas Gerais	34.00	9.60	0.79
Rio de Janeiro	27.00	10.40	0.80
São Paulo	76.00	9.00	0.84
**Brazil**	**307.00**	**11.90**	**0.77**

Sources: [1]Brazilian Institute of Geography and Statistics [32], [2]Brazilian Mortality Information System [34], [3]Industry Federation of the State of Rio de Janeiro [33]. SUS: Brazilian Unified Health System; IFDM: Firjan Index of Municipal Development.
