# Supplementary material for: Inequalities in Temporal Effects on Cervical Cancer Mortality in States in Different Geographic Regions of Brazil: An Ecological Study
Source: Int J Environ Res Public Health. 2022 May 5;19(9):5591. doi: 10.3390/ijerph19095591 (PMC9105639; doi:10.3390/ijerph19095591)
Supplement: Supplementary file 1 [file ijerph-19-05591-s001.zip › Table S3.pdf]

Table S3. Uncorrected cervical cancer mortality rates per 100,000 women in the states in the North, South, and Southeast regions in Brazil

Federation Units	1980-1984	1985-1989	1990-1994	1995-1999	2000-2004	2005-2009	2010-2014	2015-2019	SMR
South and Southeast	1980-1984	1985-1989	1990-1994	1995-1999	2000-2004	2005-2009	2010-2014	2015-2019	SMR
Paraná	8.83	9.35	10.38	11.53	10.30	8.31	7.35	7.96	8.90
Rio Grande do Sul	8.51	9.63	10.31	10.63	9.56	7.77	6.67	7.73	8.57
Santa Catarina	5.98	6.85	8.22	9.20	7.63	7.04	6.85	7.45	7.37
Espírito Santo	7.96	9.08	9.41	9.52	8.57	9.07	7.93	8.65	9.16
Minas Gerais	6.82	6.90	6.69	6.46	6.16	5.92	9.38	5.23	5.93
Rio de Janeiro	8.68	7.94	8.82	9.11	9.72	8.46	8.05	7.82	8.42
São Paulo	9.06	8.10	8.53	8.20	7.55	5.84	5.06	4.89	6.57
North	1980-1984	1985-1989	1990-1994	1995-1999	2000-2004	2005-2009	2010-2014	2015-2019	SMR
Acre	11.03	10.06	15.56	12.43	9.52	8.36	16.71	18.71	13.82
Amazonas	13.60	17.95	13.05	19.60	19.17	25.61	31.43	28.88	24.13
Amapá	20.72	40.25	28.80	22.37	17.17	15.35	23.31	20.43	21.55
Pará	14.45	14.07	12.81	11.43	13.68	14.22	13.99	16.27	14.16
Rondônia	10.46	11.84	8.43	9.66	9.42	10.86	9.54	12.01	10.44
Roraima	17.64	8.51	17.64	10.14	11.85	24.60	11.96	19.92	16.38
Tocantins	_	_	_	7.64	11.95	14.99	14.81	15.00	13.53

SMR-standardized mean mortality rate
