# Supplementary material for: Inequalities in Temporal Effects on Cervical Cancer Mortality in States in Different Geographic Regions of Brazil: An Ecological Study
Source: Int J Environ Res Public Health. 2022 May 5;19(9):5591. doi: 10.3390/ijerph19095591 (PMC9105639; doi:10.3390/ijerph19095591)
Supplement: Supplementary file 1 [file ijerph-19-05591-s001.zip › Table S4.pdf]

Table S4. Proportion of deaths from uterine cancer of unspecified portion in total uterine cancers, according to period and State

Southeast								
State	1980-84	1985-89	1990-194	1995-99	2000-04	2005-09	2010-14	2015-19
ES	39.20	41.81	41.41	30.05	31.21	21.84	32.67	20.74
MG	51.73	47.34	47.56	42.51	34.79	30.80	29.78	25.56
RJ	43.93	44.85	50.00	39.20	27.33	24.88	25.34	24.52
SP	41.05	38.52	38.74	36.42	31.10	29.73	27.52	25.79
South								
State	1980-84	1985-89	1990-194	1995-99	2000-04	2005-09	2010-14	2015-19
PR	43.30	40.62	37.31	32.17	27.33	25.70	25.37	20.49
RS	50.10	44.02	50.00	34.41	28.51	29.60	30.80	21.97
SC	48.94	43.30	40.60	33.79	32.91	29.62	25.74	21.31
North								
AC	43.30	40.62	37.31	32.17	27.33	25.70	25.37	20.49
AM	42.91	32.64	39.95	27.59	18.52	12.10	6.24	5.11
AP	43.10	7.69	23.81	31.00	28.00	36.36	9.73	14.22
PA	25.80	31.15	33.09	33.67	15.62	19.42	21.48	14.65
RO	47.30	50.72	55.24	42.26	46.72	24.79	18.68	13.14
RR	31.82	33.33	32.26	56.52	43.55	17.59	35.23	18.92
TO	—	—	—	36.51	22.11	18.61	16.33	36.51
