# Supplementary material for: Inequalities in Temporal Effects on Cervical Cancer Mortality in States in Different Geographic Regions of Brazil: An Ecological Study
Source: Int J Environ Res Public Health. 2022 May 5;19(9):5591. doi: 10.3390/ijerph19095591 (PMC9105639; doi:10.3390/ijerph19095591)
Supplement: Supplementary file 1 [file ijerph-19-05591-s001.zip › Table S5.pdf]

Table S5. Uncorrected mortality rates from cervical cancer, endometrial cancer, and uterine cancer of unspecified portion per 100,000 women in the states in the North, South, and Southeast regions in Brazil

	South							
	Period							
Locality	1980-84	1985-89	1990-94	1995-94	2000-04	2005-09	2010-14	2015-19
Paraná								
CC	8.83	9.35	10.38	11.53	10.30	8.31	7.35	7.96
EC	0.80	1.18	1.60	1.52	1.97	2.20	1.92	2.16
UCOUP	7.90	7.63	7.59	6.47	4.81	3.71	3.15	2.56
Rio Grande do Sul								
CC	8.51	9.63	10.31	10.63	9.56	7.77	6.67	7.73
EC	1.34	1.61	1.80	2.19	2.15	1.95	1.95	1.94
UCOUP	10.18	9.08	12.11	6.83	4.66	4.02	3.72	2.58
Santa Catarina								
CC	5.98	6.85	8.22	9.20	7.63	7.04	6.85	7.45
EC	0.43	0.57	1.32	1.73	1.37	1.69	1.91	1.81
UCOUP	6.61	6.28	6.80	5.87	4.73	3.76	3.06	2.45

	Southeast							
	Period							
Locality	1980-84	1985-89	1990-94	1995-94	2000-04	2005-09	2010-14	2015-19
Espírito Santo								
CC	7.96	9.08	9.41	9.52	8.57	9.07	7.93	8.65
EC	1.33	0.82	0.69	0.91	0.81	1.21	1.32	1.96
UCOUP	6.08	7.24	7.33	4.64	4.32	5.29	4.53	2.78
Minas Gerais								
CC	6.82	6.90	6.69	6.46	6.16	5.92	9.38	5.23
EC	0.58	0.68	0.82	0.80	0.99	1.05	2.70	1.81
UCOUP	8.16	7.03	6.97	5.51	3.85	3.11	5.13	2.37
Rio de Janeiro								
CC	8.68	7.94	8.82	9.11	9.72	8.46	8.05	7.82
EC	2.17	2.02	2.00	2.04	2.19	2.38	2.57	2.98
UCOUP	8.59	8.19	10.82	6.78	4.47	3.54	3.49	3.31
São Paulo								
CC	9.06	8.10	8.53	8.20	7.55	5.84	5.06	4.89
EC	1.81	1.72	2.27	2.38	2.31	2.31	2.24	2.59
UCOUP	7.80	6.01	7.01	6.21	4.51	3.46	2.74	2.54

	North							
	Period							
Locality	1980-84	1985-89	1990-94	1995-94	2000-04	2005-09	2010-14	2015-19
Acre								
CC	11.03	10.06	15.56	12.43	9.52	8.36	16.71	18.71
EC	1.23	0.00	0.00	0.99	0.64	0.87	1.60	1.91
UCOUP	9.86	6.48	7.28	6.04	4.66	3.94	2.41	1.62

Amazonas								
CC	13.60	17.95	13.05	19.60	19.17	25.61	31.43	28.88
EC	0.34	0.83	0.59	0.88	0.90	1.21	1.55	2.34
UCOUP	11.31	9.48	9.69	8.26	4.84	3.96	2.34	1.77
Amapá								
CC	20.72	40.25	28.80	22.37	17.17	15.35	23.31	20.43
EC	0.00	2.06	1.38	0.76	0.90	0.59	1.24	2.16
UCOUP	19.39	3.31	9.48	10.19	6.95	8.81	2.38	3.71
Pará								
CC	14.45	14.07	12.81	11.43	13.68	14.22	13.99	16.27
EC	0.50	0.42	0.45	0.45	0.65	0.73	1.00	1.49
UCOUP	5.80	6.90	6.69	6.42	2.79	3.75	4.20	3.11
Rondônia								
CC	10.46	11.84	8.43	9.66	9.42	10.86	9.54	12.01
EC	0.34	0.30	0.73	0.00	0.22	1.23	0.96	1.73
UCOUP	10.10	14.51	10.91	7.61	8.22	3.74	2.72	2.19
Rorraima								
CC	17.64	8.51	17.64	10.14	11.85	24.60	11.96	19.92
EC	3.47	0.00	0.00	0.77	2.15	1.92	1.13	1.96
UCOUP	12.80	3.38	8.70	15.73	10.93	5.92	7.38	5.70
Tocantins								
CC	__	__	__	7.64	11.95	14.99	14.81	15.00
EC	__	__	__	0.49	1.00	1.09	1.19	1.18
UCOUP	__	__	__	5.05	3.76	3.68	3.21	2.01

CC=cervical cancer, EC=endometrial cancer,UCOUP=uterine cancer of unspecified portion
