# Supplementary material for: Inequalities in Temporal Effects on Cervical Cancer Mortality in States in Different Geographic Regions of Brazil: An Ecological Study
Source: Int J Environ Res Public Health. 2022 May 5;19(9):5591. doi: 10.3390/ijerph19095591 (PMC9105639; doi:10.3390/ijerph19095591)
Supplement: Supplementary file 1 [file ijerph-19-05591-s001.zip › Table S6.pdf]

Table S6. Deviance and p-value analysis in sequential construction of age, period, and cohort models for the states in the South and Southeast regions of Brazil.

Models	Df[a]	Residual deviance	p (>|Chi|)
		Paraná	
Age	99	1321.65	
Age-drift[b]	98	603.75	<0.0001
Age-cohort	95	498.79	<0.0001
Age-period-cohort	92	357.69	<0.0001
Age-period	95	465.03	<0.0001
Age-drift[c]	98	603.75	<0.0001
		Rio Grande do Sul	
Age	99	1668.02	
Age-drift[b]	98	581.78	<0.0001
Age-cohort	95	557.60	<0.0001
Age-period-cohort	92	402.35	<0.0001
Age-period	95	436.75	<0.0001
Age-drift[c]	98	581.78	<0.0001
		Santa Catarina	
Age	99	449.29	
Age-drift[b]	98	337.92	<0.0001
Age-cohort	95	288.56	<0.0001
Age-period-cohort	92	222.17	<0.0001
Age-period	95	272.83	<0.0001
Age-drift[c]	98	337.92	<0.0001
		Espírito Santo	
Age	99	399.26	
Age-drift[b]	98	257.97	<0.0001
Age-cohort	95	247.32	<0.0001
Age-period-cohort	92	185.04	<0.0001
Age-period	95	194.75	<0.0001
Age-drift[c]	98	257.97	<0.0001
		Minas Gerais	
Age	99	1675.28	
Age-drift[b]	98	630.99	<0.0001
Age-cohort	95	543.58	<0.0001
Age-period-cohort	92	493.70	<0.0001
Age-period	95	570.67	<0.0001
Age-drift[c]	98	630.99	<0.0001
		Rio de Janeiro	
Age	99	1453.96	
Age-drift[b]	98	900.29	<0.0001
Age-cohort	95	735.40	<0.0001
Age-period-cohort	92	498.60	<0.0001
Age-period	95	764.12	<0.0001
Age-drift[c]	98	900.29	<0.0001
		São Paulo	

Age	99	6131.26	
Age-drift[b]	98	2072.19	<0.0001
Age-cohort	95	1811.57	<0.0001
Age-period-cohort	92	1523.47	<0.0001
Age-period	95	1770.21	<0.0001
Age-drift[c]	98	2072.19	<0.0001

[a]Degrees of freedom; [b]linear trend for the logarithm of age-specific rates, which is equal to the sum of the period and cohort slopes ($\beta L + \gamma L$), where βL and γL are the period and cohort linear trends, respectively; [c]longitudinal age trend, which is the sum of age and period slopes ($\alpha L + \beta L$), where αL and βL are the age and period linear trends, respectively.
