# Supplementary material for: Inequalities in Temporal Effects on Cervical Cancer Mortality in States in Different Geographic Regions of Brazil: An Ecological Study
Source: Int J Environ Res Public Health. 2022 May 5;19(9):5591. doi: 10.3390/ijerph19095591 (PMC9105639; doi:10.3390/ijerph19095591)
Supplement: Supplementary file 1 [file ijerph-19-05591-s001.zip › Table S7.pdf]

Table S7. Deviance and p-value analysis in sequential construction of age, period, and cohort models for the states in the North Region in Brazil.

Models	Df[a]	Residual deviance	p (>|Chi|)
Acre			
Age	99	175.22	
Age-drift[b]	98	172.68	0.11
Age-cohort	95	171.93	0.86
Age-period-cohort	92	158.85	0.007
Age-period	95	160.97	0.77
Age-drift[c]	98	172.68	0.008
Amazonas			
Age	99	299.94	
Age-drift[b]	98	258.53	<0.0001
Age-cohort	95	233.02	<0.0001
Age-period-cohort	92	200.30	<0.0001
Age-period	95	228.09	<0.0001
Age-drift[c]	98	258.53	<0.0001
Amapá			
Age	99	172.27	
Age-drift[b]	98	146.30	<0.0001
Age-cohort	95	143.85	<0.0001
Age-period-cohort	92	138.02	<0.0001
Age-period	95	140.98	<0.0001
Age-drift[c]	98	146.30	<0.0001
Pará			
Age	99	315.62	
Age-drift[b]	98	312.17	<0.0001
Age-cohort	95	296.00	<0.0001
Age-period-cohort	92	268.60	<0.0001
Age-period	95	289.28	<0.0001
Age-drift[c]	98	312.17	<0.0001
Rondônia			
Age	99	226.845	
Age-drift[b]	98	167.07	<0.0001
Age-cohort	95	156.78	<0.0001
Age-period-cohort	92	152.00	<0.0001
Age-period	95	161.79	<0.0001
Age-drift[c]	98	167.07	<0.0001
Roraima			
Age	99	159.83	
Age-drift[b]	98	159.40	0.52
Age-cohort	95	158.24	0.76
Age-period-cohort	92	144.89	0.003
Age-period	95	145.29	0.94

Age-drift[c]	98	159.40	0.004
Tocantins			
Age	60	103.83	
Age-drift[b]	59	102.91	0.33
Age-cohort	56	95.20	0.05
Age-period-cohort	54	86.60	0.01
Age-period	57	94.48	0.05
Age-drift[c]	59	102.91	0.01

[a]Degrees of freedom; [b]linear trend for the logarithm of age-specific rates, which is equal to the sum of the period and cohort slopes ($\beta L + \gamma L$), where βL and γL are the period and cohort linear trends, respectively; [c]longitudinal age trend, which is the sum of age and period slopes ($\alpha L + \beta L$), where αL and βL are the linear age and period trends, respectively.
